# Autophagy in Hepatic Fibrosis

**DOI:** 10.1155/2014/436242

**Published:** 2014-03-23

**Authors:** Yang Song, Yingying Zhao, Fei Wang, Lichan Tao, Junjie Xiao, Changqing Yang

**Affiliations:** ^1^Division of Gastroenterology and Hepatology, Digestive Disease Institute, Shanghai Tongji Hospital, Tongji University School of Medicine, 389 Xincun Road, Shanghai 200065, China; ^2^Regeneration Lab and Experimental Center of Life Sciences, School of Life Science, Shanghai University, 333 Nan Chen Road, Shanghai 200444, China; ^3^Department of Cardiology, The First Affiliated Hospital of Nanjing Medical University, Nanjing 210029, China; ^4^Shanghai Key Laboratory of Bio-Energy Crops, School of Life Science, Shanghai University, Shanghai 200444, China

## Abstract

Hepatic fibrosis is a leading cause of morbidity and mortality worldwide. Hepatic fibrosis is usually associated with chronic liver diseases caused by infection, drugs, metabolic disorders, or autoimmune imbalances. Effective clinical therapies are still lacking. Autophagy is a cellular process that degrades damaged organelles or protein aggregation, which participates in many pathological processes including liver diseases. Autophagy participates in hepatic fibrosis by activating hepatic stellate cells and may participate as well through influencing other fibrogenic cells. Besides that, autophagy can induce some liver diseases to develop while it may play a protective role in hepatocellular abnormal aggregates related liver diseases and reduces fibrosis. With a better understanding of the potential effects of autophagy on hepatic fibrosis, targeting autophagy might be a novel therapeutic strategy for hepatic fibrosis in the near future.

## 1. Introduction

Hepatic fibrosis, a leading cause of morbidity and mortality worldwide, is usually associated with chronic liver diseases caused by infection, drugs, metabolic disorders, or autoimmune imbalances. Hepatic fibrosis can develop into cirrhosis within 1–10 years with a 7- to 10-year liver-related mortality of 12% to 25% [1]. Unfortunately, effective clinical therapies are still lacking.

Hepatocytes have a dramatic regenerative capability. Usually, the necrotic or apoptotic hepatocytes are replaced through the replication of adjacent hepatocytes within the lobules. However, under heavy and sustained damage, the regenerative capability of hepatocytes will be impaired, and, consequently, hepatic stellate cells are activated, inducing liver fibrogenesis. Autophagy, a catabolic process by which cells develop, differentiate, survive, and stay homeostasis under conditions such as nutrients deprivation and hypoxia, has been implicated in many liver diseases including viral hepatitis, alcohol liver diseases, nonalcohol liver diseases, acute liver injury, and alpha1-antitrypsin (AT) deficiency related liver diseases [2–5]. As hepatic fibrosis is a common outcome of a variety of chronic liver diseases, this review will highlight and summarize recent progresses of the role of autophagy in hepatic fibrosis.

## 2. Autophagy

Autophagy, a metabolic process that eukaryotic cells digest their own organelles and long-lived proteins, is critical for development, differentiation, and homeostasis. It is the only way that “old” or “broken” organelles degrade [6, 7]. As a necessary process to maintain cell survival during starvation and damage, the proteins involved in autophagy are highly conserved from yeast to mammalian.

In the initial stage, a membranal structure named phagophore, will extend and sequester cytoplasmic organelles which includes mitochondria, endoplasmic reticulum, and ribosomes. After that, the edges of the membrane fuse to form a double-deck membranal spherical structure, autophagosome. Later, it fuses with lysosome and delivers the inner membrane and its inclusion to lysosome. The fusion of autophagosome and lysosome is called autolysosome, and it is where the cargoes are degraded, and then hydrolyzed products are transported to plasma for cellular energy recycling [8]. The deficiency or the mutation of related genes leads to abnormal protein aggregates, immunity deficiency, and oncogenesis. PI3K/Akt/mTOR signaling pathway is a major contributor for autophagy. Under deprivation of nutrients or growth factors mTOR signaling is inhibited, which thereby induces autophagy [9]. Moreover, while nutrients and growth factors are abundant, activation of mTORC1 suppresses the ULK1 complex and autophagy and promotes cell growth and metabolic activity. In mammal, class III PI3K can stimulate autophagy and form complexes with p150 and Beclin1, which is regulated by many molecules including UVRAG, Bif-1, Rubicon, and Ambra1 [10]. However, class I PI3K inhibits autophagy by activating mTOR and PKB [11]. Autophagy is also regulated by Ras/PKA signaling pathway in addition to PI3K/Akt/mTOR signaling pathway. Inactivation of PKA by a dominant negative allele of Ras2 can induce autophagy even under nutrient-rich conditions [12].

As a cellular housekeeper, autophagy eliminates defective proteins and organelles, removes intracellular pathogens, and also prevents abnormal proteins from accumulating. Therefore, autophagy plays an active role in the pathology of many diseases, including cancer [13], infection [14, 15], neurodegeneration [16], aging [17], and cardiovascular diseases [18]. In neurodegenerative diseases, the accumulation of autophagosome is observed, which is resulted from the inhibition of the fuse between autophagosome and lysosome [16]. Loss of autophagy induces the accumulation of abnormal proteins, contributing to neurodegenerative diseases including Alzheimer's disease, transmissible spongiform encephalopathies, Parkinson's disease, and Huntington's disease [16, 19, 20]. Similarly, Danon diseases, characterized by cardiomyopathy and myopathy, are associated with the failure of autophagosome to fuse with lysosome. Moreover, tumor suppressor genes including PTEN, TSC1, and TSC2 that stimulate autophagy, are inhibitors of mTOR signaling in the upstream. Conversely, mTOR-activating oncogene products such as class I PI3K and Akt inhibit autophagy. P53, which often mutates in human cancers, regulates autophagy through the mTOR pathway [21, 22]. Constitutive activation of the PI3K pathway is among the most common events in human cancer, and the downstream kinase mTOR restricts autophagy in response to starvation [23, 24].

## 3. Autophagy: An Important Player in Tissue Fibrosis 

Cystic fibrosis (CF), a genetic disease relatively occurring more frequently in Caucasians [25–27], is clinically characterized by chronic severe lung inflammation. Cystic fibrosis transmembrane conductance regulator (CFTR) is considered as one molecular regulator of CF [26], and a sequestration of misfolding of CFTR has been observed in airway epithelia of CF patients. CFTR defection inhibits autophagy, and rescuing autophagy could favor the clearance of the aggresomes and attenuates inflammation in CF both in vivo and in vitro [28]. Similarly, rapamycin, an autophagy inducer, suppresses lung inflammation and infection by* Burkholderia cenocepacia* [29]. In addition, azithromycin, a blocker of autophagy, leads to mycobacterial infection in CF patients [30]. The above data consistently supports the idea that autophagy is critically involved in CF. Unilateral ureteral obstruction (UUO) is a classical model of progressive renal fibrosis. Autophagy is induced in obstructed kidney after UUO induction, and inhibition of autophagy by 3-MA enhances tubulointerstitial fibrosis, indicating a renoprotective role of autophagy in renal fibrosis [31, 32]. In a TGF-beta overexpression transgenic mouse model which exhibits widespread peritubular fibrosis, tubular cells decomposition is induced by autophagy [33].

Being a center player in fibrosis, autophagy is involved in almost all fibrosis related diseases within diverse organs or systems. This review will focus on hepatic fibrosis, a common pathological process occurring in most chronic liver diseases.

## 4. Mechanisms of Hepatic Fibrosis

Hepatic fibrosis, a scarring of wounded liver, is a process in which liver compensates its loss of parenchyma cells through fibrogenesis. In Western societies, alcohol abuse is the major cause of liver fibrosis [34, 35], while virus infection especially HBV and HCV dominates in Asian and African countries [36, 37]. Some drugs or chemicals have also been proved to cause hepatic fibrosis. For example, chronic hepatic inflammation and fibrogenesis have been identified in patients who accepted long-term, low-dosage paracetamol administration [38]. On the other hand, some chemicals such as tetrachloromethane and N-nitrosodimethylamine were used as well-established hepatotoxic reagents to induce hepatic fibrosis in rodents [39, 40]. Many other etiological factors of hepatic fibrosis have also been well-described over the past decades, including alcohol abuse, nonalcoholic steatohepatitis, autoimmune hepatitis, schistosomiasis, and metabolism disorder [41, 42].

Although acute injury will also activate the process of fibrogenesis, liver injury associated with chronic liver diseases is required for significant fibrosis to accumulate. Any chronic perturbation of hepatic homeostasis, whether visible by light microscopy or not, may elicit the signals necessary for the initiation of fibrogenesis [41]. Chronic liver injury, regardless of etiology, induces liver fibrogenesis through a dynamic and highly integrated process that leads to progressive accumulation of extracellular matrix (ECM) components with an attempt to limit hepatic damage. Sustained liver injury activates resident hepatic stellate cells (HSCs), which are considered as a major source of fibrogenesis though portal fibroblast [43], bone marrow derived fibrocytes [44], and resident hepatocytes undergoing epithelial to mesenchymal transition [45] may also contribute to hepatic fibrogenesis [46].

**Figure 1:**
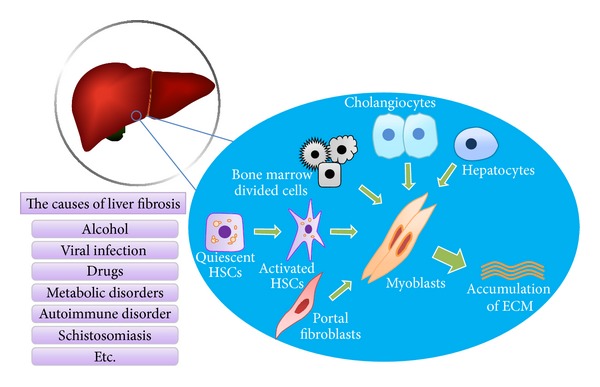
Essential processes of hepatic fibrosis.

HSCs are the major cells producing ECM [47]. In normal liver, HSCs reside in the space of Disse. Upon injury, HSCs are activated and transdifferentiated into myofibroblast-like cells. Activated HSCs migrate and accumulate at the sites where tissue is impaired, producing large amount of ECM and reducing ECM from degradation (Figure 1). Activation of TGF-beta signaling [48, 49], PDGF, or other growth factors [50, 51] and oxidative stress [52] have been identified to contribute to the activation of HSCs. Activation of HSCs is composed of two phases: initiation and perpetuation [53]. The most informative feature that HSCs are undergoing initiation phase is the transformation from LD-rich cells to myofibroblast-like cells [54], which is accompanied with increased autophagy flux [55]. This morphological change suggests that autophagy may function in the process of HSCs activation associated with LDs mediated pathway.

## 5. Autophagy in Fibrogenic Cells

HSCs are well-known as major fibrogenic cells in liver and are filled with cytoplasmic LDs before being activated. LDs are neutral lipid storage organelles that are found in all organisms from bacteria to human [56]. As LDs are internal nutrient stores for use during starvation, their contents are accessed primarily through the actions of specific enzymes, such as hormone-sensitive lipase. LC3 conjugation system, which is important in the process of autophagy [57], is critically involved in the formation and degradation of LDs.

Quiescent HSCs are filled with cytoplasmic LDs containing retinyl esters (especially retinyl palmitate) and triacylglycerols, accounting for more than 70% of their lipid content [58, 59]. Upon activation of the HSCs, LDs reduce in size while increase in number in the initial phase, and LDs migrate to cellular extensions before they disappear [60]. Along with the switch from LDs-rich cells to myofibroblast-like cells, autophagy flux is upregulated [55]. It has been demonstrated that cellular lipids stored as triglycerides in LDs would be hydrolysed into fatty acids for energy [61]. Inhibition of autophagy increases triglyceride storage in LDs [62]. Autophagy may supply energy for activation of HSCs by delivering triglyceride and other components in LDs from autophagosomes to lysosomes for degradation.

The number and size of LDs are consistently increased in HSCs treated with autophagy inhibitor 3MA [63] or knocked out the autophagy related gene Atg5. Moreover, the rate of *β*-oxidation, which indicates the levels of FFA generated by triglyceride hydrolysis [64], increases during lipid loading, but to a much lesser extent in cells with inhibited autophagy [62, 65]. Based on these evidences, we could hypothesize that autophagy may be the energy supplier for HSCs activation and lipids (mainly triglyceride) contained in LDs as fuel [66]. A recent study has showed that nilotinib could induce cell death of HSCs, and inhibiting apoptosis alone did not reduce HSCs death because autophagic cell death was exacerbated [67]. This effect was only found in activated HSCs but not quiescent HSCs, indicating that autophagy may have different function in activated HSCs and quiescent HSCs. Moreover, 3-MA induced autophagy inhibitory was reported to cause an arrest in the G2 phase of HSC-T6 cells, a rat HSC line, and thereafter inhibited the proliferation of HSC-T6 cell [68], suggesting that autophagy is required for HSCs proliferation besides affecting LDs metabolism as described elsewhere.

Resident HSCs are not the only source of myofibroblast which contributes to fibrogenesis in liver. To date, several other types of cells have been proved to be involved in hepatic fibrosis, including portal fibroblasts, circulating fibrocytes, bone marrow derived fibrocytes, and hepatocytes via an EMT (epithelial-mesenchymal transition) program. Although relatively little information is known about autophagy and EMT in hepatocytes, other studies in other diseases might also provide some implications. Recent studies have found that DEDD, a novel tumor repressor, could activate selective autophagy and thereafter induce the degradation of Snail and Twist, two master regulators of EMT in human breast cancer [69, 70]. Another study found that starvation-induced autophagy could induce the expression of EMT markers and invasion in hepatic carcinoma cells through a TGF-*β*/Smad3 signaling-dependent manner, and the inhibitory of autophagy in the starvation could result in the suppression of EMT [71]. These results give potential indirect evidence that autophagy might also have potential effects on EMT of hepatocytes and thus participates in the process of hepatic fibrosis.

## 6. Autophagy, Associates in Crime?

Not only can autophagy act as an energy fueler for HSCs activation, it can as well induce some liver diseases to develop inducing hepatic fibrogenesis.

Viral hepatitis, a world-widespread public health concern, can be caused by several viruses. Among them, hepatitis B virus (HBV) and hepatitis C virus (HCV) are two major types. HCV is a single, positive-stranded membrane-enveloped RNA virus, belonging to the* Hepacivirus* genus in the Flaviviridae family. HCV infection induces autophagy despite viral genotype. Autophagy is increased in cells harboring HCV strains including H77 (genotype 1a), Con1 (genotype 1b), and JFH1 (genotype 2a) [72–74], which is associated with endoplasmic reticulum (ER) stress [74]. HCV induces early phase of autophagy in hepatocytes, with the accumulation of autophagosomes and the upregulation of the ratio of LC3-II to LC3-I [72, 74]. In addition, HCV uses autophagy pathway for its own replication. Virus-induced unfolded protein response (UPR) may activate autophagy to support the virus life cycle. As virus proliferates, the expression of HCV core and nonstructural proteins induce HSCs to proliferate and inflammatory cytokines to be secreted. In addition, hepatocytes harboring and replicating HCV in culture produce fibrogenic stimuli towards HSCs [75, 76]. Interestingly, autophagic protein are only required at the beginning phase at which incoming HCV RNA transfected to the cell translation apparatus, but the HCV RNA was observed not collocated with HCV core and nonstructural proteins. Downregulation of autophagic proteins 10 days after transduction does not affect HCV replication, suggesting that autophagic proteins are not necessary for HCV replication once established [73]. On the other hand, HCV infection has been suggested to impair the late stage of autophagic pathway by inhibiting the maturation of autolysosome, as the observed extensive aggregation long-lived protein p62 and the insufficient mature autophagic vacuoles in HCV harboring cells [74]. Besides HCV, autophagy is required for HBV replication. Inhibition of autophagy with 3-methyladenine (3-MA) markedly inhibited the production of HBV [77]. HBV induced autophagy is associated with HBV x protein (HBx) and HBV small surface protein (SHBs) [77, 78]. However, how HBV uses autophagy for its own replication remains unclear.

Primary biliary cirrhosis (PBC) is a form of liver disease that over time can lead to liver cirrhosis [79]. LC3, an autophagy maker, is more frequently in bile ductular cells of both early stage and advanced stage of PBC patients than that in control groups. LC3 is significantly correlated with the expression of cellular senescence makers, suggesting that autophagy may be involved in cellular senescence in PBC. Given the idea that cellular senescence is involved in ductular reaction (DR) in primary biliary cirrhosis [80], autophagy may be a novel player in PBC. However, it is unclear whether this upregulated autophagy is protective or harmful, and studies revealing the underlying molecular mechanism are also scarce.

## 7. Autophagy May Protect Hepatocellular Abnormal Aggregates Related Liver Diseases and Reduce Fibrosis

Besides the activation of HSCs, chronic hepatocyte injury is another key step for hepatic fibrogenesis. It has been demonstrated that autophagy was involved in many liver diseases with abnormal hepatocellular aggregates, such as alcohol/nonalcohol steatohepatitis and alpha1-antitrypsin (AT) deficiency liver disease. Autophagy is considered as a protective factor that overcomes hepatocellular protein aggregation burdens, which has been observed in the liver diseases above and these burdens, like AT Z protein, may induce liver injury.

AT deficiency, caused by homozygosity for the AT mutant Z gene (ATZ), is clinically characterized by liver disease and early-onset emphysema, which affects one in 2000–5000 individuals [81]. AT matures in the endoplasmic reticulum (ER), while in the classical form of AT deficiency, a point mutation in AT alters the folding of a liver-derived secretory glycoprotein in hepatocytes. Polymers of ATZ, those being normally cleared from the ER via the autophagic pathway, have been identified by electron microscopy as diastase-resistant inclusions within the ER of hepatocytes [82]. When polymers of ATZ accumulate in the ER, they can be degraded by two pathways, the proteasome and autophagy pathways [83]. The former probably aims at the soluble forms of ATZ [84], while the latter may focus on the polymerized forms of ATZ [85]. As the basic or impaired autophagy is not able to match the upregulation of ATZ aggregates formation, ATZ is prone to aggregate in ER, leading to subsequent ER stress, hepatocytes death, and liver injury [86]. In AT deficiency patients and ATZ transgenic animal model, autophagosome has been reported to be increased in number [85]. In human hepatoma cell lines and fibroblast cell lines overexpressing ATZ, an increased colocation of autophagosomes and ATZ aggregates has been observed. Meanwhile, inhibition of autophagy in these cell lines leads to the accumulation of misfolded ATZ in ER and worsens liver injury [87]. Carbamazepine, an autophagy-enhancing drug, promotes the degradation of ATZ polymers and then reduces the level of hepatic fibrosis [88]. In addition, liver-directed gene transfer of transcription factor EB (TFEB), a major regulator of lysosomal function and autophagy, prevents hepatocytes from apoptosis and fibrogenesis [89]. Moreover, upregulated autophagy by rapamycin effectively reduces ATZ aggregation in hepatocytes with the reduction of hepatocellular injury makers and the level of hepatic fibrosis [90]. These findings suggest that autophagy plays a protective role in the pathology of AT deficiency liver disease and reduces hepatic fibrosis.

Chronic alcohol abuse leads to hepatic lesions such as alcoholic hepatitis, hepatic fibrosis, and cirrhosis. Mallory-Denk bodies (MDBs), found in the livers of alcohol hepatitis and alcohol cirrhosis patients [91, 92], are mainly made up of keratins 8 and 18 (K8/18), ubiquitin, and p62 [92, 93]. Autophagy is involved in the elimination of MDBs in hepatocyte, and the accumulation of MDBs may be an evidence of the decrease of autophagy in alcoholic hepatitis [94]. In addition, in the hepatocytes of Atg7-deficient mice, MDB-like protein aggregates are observed [95], indicating that autophagy deficiency may lead to abnormal protein aggregates formation and liver injury in alcoholic hepatitis. Autophagy inhibition is able to increase steatosis in animal models of alcohol induced hepatic injury [96]. MDBs are not limited in alcoholic hepatitis and are now believed as a recognized feature of many other liver diseases including nonalcoholic steatohepatitis (NASH). NASH is characterized by abnormal lipid metabolism and the accumulation of TGs storage in LDs of hepatocytes, and this accumulation of lipids contributes to the initiation of NASH. By inducing starvation in vivo, mice livers show autophagic maker LC3 associated with LDs and the presence of lipid in autophagosomes and lysosomes, indicating that autophagy may be an important pathway that mediates lipo-degradation [65]. Low levels of autophagy or impaired autophagic flux may be a potential risk factor that exacerbates steatosis and the subsequent fibrosis by promoting both the initial lipid accumulation and the progression to cellular injury [97].

In an autophagy-deficient yeast chain, secretory protein shows stabilization of aggregated ER form, indicating that autophagic pathway is a conservative process to remove abnormal aggregation from ER [98]. However, how these misfolded proteins are recognized and removed by the autophagy pathway is unclear [99] though unfolded protein response (UPR) pathway, JNK pathway, and PERK pathway have been reported to be involved in that [100–103]. Autophagic target p62 can be detected in both MBs, and it aggregates in the ATZ liver, suggesting that it can be subjected to autophagic removal. P62 molecule is common in these inclusion bodies and it can be a bridge for misfolded proteins and autophagosome by binding to misfolded proteins and LC3 on autophagosome's membrane [104]. This was further confirmed by studies in ATZ liver diseases [88, 89].

## 8. Conclusion

Hepatic fibrosis is a common pathological process that is involved in most chronic liver diseases. The advanced stage of hepatic fibrosis named cirrhosis is highly deadly and is often accomplished with multiple complications and hepatic function disorders. Recent studies have demonstrated that hepatic fibrosis was reversible, suggesting that therapy targeting hepatic fibrogenesis is feasible. Autophagy induces the activation of HSCs, a key process for the genesis of hepatic fibrosis. In addition, autophagy plays diverse roles in liver diseases that cause hepatocellular damage and subsequent fibrogenesis. However, the profibrosis effect of autophagy is mainly carried out in resident HSCs; according to recent studies, seldom evidences were found that autophagy is regulated in other fibrogenic cells. Moreover, as autophagy is protective in most cells, nonspecific antiautophagy therapies may result in many unwanted effects. Thus, antiautophagy regents with highly specific affinity to HSCs may be novel therapeutic strategy for hepatic fibrosis in the near future.
